# Transient heat release during induced mitochondrial proton uncoupling

**DOI:** 10.1038/s42003-019-0535-y

**Published:** 2019-07-26

**Authors:** Manjunath C. Rajagopal, Jeffrey W. Brown, Dhruv Gelda, Krishna V. Valavala, Huan Wang, Daniel A. Llano, Rhanor Gillette, Sanjiv Sinha

**Affiliations:** 1 Department of Mechanical Science and Engineering, University of Illinois at Urbana-Champaign, Urbana, IL 61801 USA; 2 College of Medicine, University of Illinois at Urbana-Champaign, Urbana, IL 61801 USA; 3 Re3 Innovative Neuroscience Institute, Sarasota, FL USA; 4 Department of Molecular and Integrative Physiology, University of Illinois at Urbana-Champaign, Urbana, IL 61801 USA

**Keywords:** Cell biology, Sensors and probes

## Abstract

Non-shivering thermogenesis through mitochondrial proton uncoupling is one of the dominant thermoregulatory mechanisms crucial for normal cellular functions. The metabolic pathway for intracellular temperature rise has widely been considered as steady-state substrate oxidation. Here, we show that a transient proton motive force (pmf) dissipation is more dominant than steady-state substrate oxidation in stimulated thermogenesis. Using transient intracellular thermometry during stimulated proton uncoupling in neurons of *Aplysia californica*, we observe temperature spikes of ~7.5 K that decay over two time scales: a rapid decay of ~4.8 K over ~1 s followed by a slower decay over ~17 s. The rapid decay correlates well in time with transient electrical heating from proton transport across the mitochondrial inner membrane. Beyond ~33 s, we do not observe any heating from intracellular sources, including substrate oxidation and pmf dissipation. Our measurements demonstrate the utility of transient thermometry in better understanding the thermochemistry of mitochondrial metabolism.

## Abstract (web summary)

Rajagopal et al. show that transient, intracellular temperature spikes coincide well with electrical heating from proton transport across the mitochondrial membrane in *Aplysia* neurons. A lack of sustained temperature rise suggests that a transient dissipation of proton motive force rather than substrate oxidation may play a dominant role for heat generation.

## Introduction

**Fig. 1:**
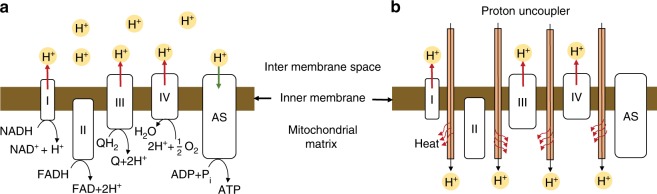
Proton uncoupler in action at the mitochondrial inner membrane. **a** A schematic of the mitochondrial respiratory chain shows three protein complexes (I, III, IV) producing an H^+^ gradient across the inner mitochondrial membrane. ATP synthase (AS) utilizes this H^+^ gradient to synthesize ATP from ADP. **b** Proton uncouplers allow diffusion of protons through the mitochondrial membrane. This sudden diffusion into the mitochondrial matrix results in a proton current that can generate heat

Biochemical reactions are sensitive to variations in temperature, pH, O_2_, glucose, etc., of which temperature has been the focus of several studies by clinical neuroscientists^1^. Temperature fluctuations in the brain on the order of 1–2 °C can impact memory encoding, effect behavioral changes, and generate autonomic responses^1^. The apparent sensitivity to overall brain temperature originates from reactions at the level of individual neurons. To counteract large external temperature fluctuations, animal cells have evolved certain thermoregulatory mechanisms. For instance, heat shock has been shown to trigger compensatory intracellular endothermic reactions^2^ that can alter gene expressions and activate signaling cascades^3^. On the other hand, in adapting to cold environments, for instance, exothermic non-shivering thermogenesis is induced at mitochondria^4–6^ to produce heat. Despite recognition of the fundamental involvement of temperature in eliciting biochemical changes, specific molecular mechanisms for heat evolution in cells are still not clearly identified experimentally^7–10^. It is widely known that biological uncoupling proteins (UCP) uncouple oxidative phosphorylation, thereby converting the energy required to synthesize ATP into heat^11,12^. This steady-state substrate oxidation is expected to produce only ~10^−5^ K temperature increase per cell^7–10^. However, at the onset of proton uncoupling (Fig. 1), a transient proton motive force (pmf) dissipation occurs before enhancing substrate oxidation. In this work, we experimentally demonstrate for the first time that chemically induced pmf dissipation can result in large intracellular temperature spikes of ~4.8 K over a short duration of ~1 s in *Aplysia* neurons.

Proton uncoupling has been hypothesized to result in transient electrical heating^13^ (Fig. 1b). The electrochemical proton motive force (Δ*p*) generated by proton pumps (Fig. 1a) are typically 150–200 mV^13–15^. By sharply dissipating the whole mitochondrial potential (~150 mV) using a patch clamp, previous studies^16,17^ reported an exponentially decreasing proton current ($I_{{\rm{H}}^ {+}}$) with a maximum current ~150 pA (Supplementary Fig. 1)^16^. The resulting heat ($\dot Q\sim {\mathrm{\Delta }}p \cdot I_{{\rm{H}}^ {+}}$) can cause a temperature rise in a single mitochondrion at a rate of ~4.8 K/s at the onset of proton motive force dissipation^13^. This heat pulse is expected to be <1 s owing to the short duration of proton currents (Supplementary Fig. 1)^16^. We note that the previously reported magnitudes of $I_{{\rm{H}}^ {+}}$ and Δ*p*, and the duration of proton current may vary across different cell lines depending on the expression of UCP^17^ and the proton pool. However, irrespective of UCP expression, chemical proton uncouplers can similarly increase the permeability^18^ of protons across the inner membrane of mitochondria, resulting in a short-lived proton motive force dissipation and associated heating. Previous thermometry on proton uncoupling probed longer temporal scales of ~5 min or more^19–24^ but missed information on short-term pmf dissipation effects. To record transients during pmf dissipation, a thermometry technique that combines low thermal time constant (<1 s) with high accuracy (<±1 K), as well as chemical and electrical inertness, is necessary.

Previous reports of non-invasive thermometry^19,22,25^ using temperature-dependent fluorescence lifetimes or intensities typically had accuracies^26^ ≳1 K. They also suffered from off-target signals^7,22,27^ that came from photobleaching^22^, variations in microscale viscosity, ion concentrations, and intracellular pH changes within the cellular environment^7^. Chemically inert micro-fabricated thermocouples^28^ were previously made that measured extracellular, but not intracellular temperatures. Invasive intracellular thermometers have been made from micropipettes^29–32^ and tungsten-based probes^33,34^. Metal-filled micropipettes^31^ have a thermal time constant ~0.6 s, which is high for measuring transient pmf dissipation over <1 s. Tungsten probes that have a long (7–10 μm) junction^33^ measure spatially averaged temperatures inside a cell. Invasive thermometers that are also electrically bare^29–34^ suffer from common mode noise^35^, when used in an electrically active cellular milieu. Moreover, previous reports^29–34^ typically used a water bath for calibrating the temperature response of the sensors. This can result in errors arising from local convection effects, and temperature differences between reference sensor and the probe. Overall, existing sensing techniques lack the required chemical and electrical inertness, accuracy (<±1 K), and low thermal time constant (<1 s) to measure transient pmf dissipation.

**Fig. 2:**
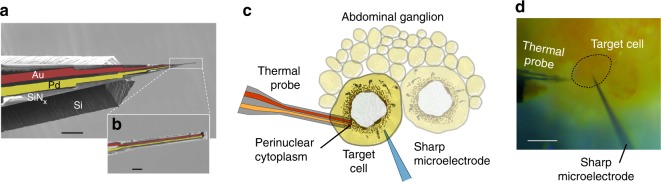
Schematic and microscopy images of intracellular temperature measurement inside *Aplysia* neurons. **a** A false-colored scanning electron microscopy image of the thermal probe. The suspended region is ~451-μm long. Scale bar corresponds to 100 μm. **b** The temperature-sensitive thermocouple junction is ~1 μm in diameter. Scale bar corresponds to 5 μm. **c** A schematic of the setup used for measuring temperature changes inside the cell while concurrently monitoring the membrane potential using a KCl sharp microelectrode. The brown patches in the perinuclear cytoplasm are representative of mitochondrial sites in *Aplysia* neurons^38^. **d** An optical image of the abdominal ganglion of *Aplysia*. The two probes are inside the target cell R15. Scale bar corresponds to 100 μm

Here, we employed a microscale thermocouple probe to capture such transients in intracellular temperatures. Fig. 2a, b show the probe, fabricated using the techniques of silicon-based microelectromechanical systems (MEMS). Details of the fabrication are explained in Supplementary Fig. 2 and in our previous work^26^. We performed an on-chip calibration in a vacuum cryostat (Supplementary Fig. 2e), and determined the calibration accuracy to be ~±54 mK at 300 ± 10 K. We note that we do not use a heated culture medium, as was done in previous studies^29–34^, for calibrating the thermal probe. However, we tested our probe in a heated culture medium to confirm the temperature response (Supplementary Fig. 3). The temperature-sensitive Au/Pd thermocouple junction is 1 μm in diameter. It is supported by a 1-μm-thick silicon nitride cantilever tip of 5 μm width. We calculated the time constant of the probe^26^ to be 32 μs. The probe is electrically insulated with ~300 nm of silicon nitride. In Supplementary Fig. 4, we show that the probe is insulated from common mode signals, resulting in <20 mK noise in a typical electrically active neuron.

We made intracellular measurements on neurons from the sea slug *Aplysia californica*. The animal’s abdominal ganglion, which constitutes parts of a distributed central nervous system, possesses neurons that can reach up to ~1 mm in diameter^36^, with nuclei^37^ as large as ~800 μm. The perinuclear cytoplasm is enriched with mitochondria^38,39^. The *Aplysia* neurons found superficially in the abdominal ganglion are typically hundreds of microns in diameter, which renders them favorable to penetration with our thermal probe that is ~5-μm wide. Unless mentioned otherwise, we used neurons from the abdominal ganglia throughout the study. We also utilized a sharp voltage microelectrode to record the real-time membrane potential of the neuron as a metric of cell health (Fig. 2c). More details on the microelectrode and culture dish preparation are in the Methods section. Figure 2d shows an optical microscope image of the culture dish with thermal probe and voltage microelectrode inside a neuron.

In this work, we measure transient intracellular temperature changes during proton motive force dissipation, which is induced by chemical proton uncouplers. We first identify off-target responses to the proton uncouplers. Then, we extract intracellular temperature responses from proton motive force dissipation.

## Results

### Identifying suitable reagents

**Fig. 3:**
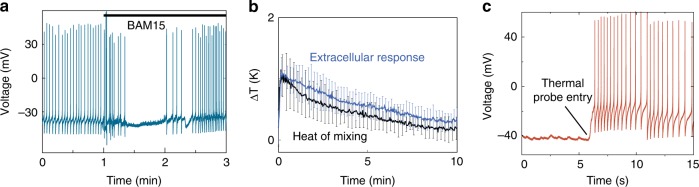
Characterizing intracellular and extracellular responses. **a** Representative plot of membrane potential before, during, and after the addition of 10 µM of the proton uncoupler BAM15 (*n* = 6). The neuron was penetrated by the thermal probe before the addition of BAM15. **b** Control experiments with BAM15 show a maximum extracellular temperature rise of 1.2 ± 0.6 K that decays over ~8 min. Extracellular responses were measured with the thermal probe placed just outside the cell membrane of a target neuron (*n* = 6). Heat of mixing between BAM15 and saline was measured in the absence of a ganglion (*n* = 6). **c** A representative injury discharge (*n* = 6) that is observed as the thermal probe enters the cell. All the intracellular data plotted in Fig. 4 are from experiments that recorded a positive injury discharge response, indicating the probe’s presence inside the cell

Carbonyl cyanide m-chlorophenyl hydrazine (CCCP) is a widely known protonophore^18,40^ that is also a positive control^20,22^ for eliciting a temperature rise inside a cell (Supplementary Fig. 5). However, CCCP produces undesirable off-target^41–43^ effects including cytotoxicity, which manifests in neurons as rapid membrane depolarization^42^ (Supplementary Fig. 5). In order to observe temperature changes that originate from proton motive force dissipation, we instead choose BAM15 (5-N,6-N-bis(2-fluorophenyl)-[1,2,5]oxadiazolo[3,4-b]pyrazine-5,6-diamine)^41^ to uncouple protons. Unlike CCCP, BAM15 does not exert the same degree of off-target effects^41^. This is consistent with our observation of unperturbed neuronal membrane potential (Fig. 3a), which is also a confirmation that mitochondrial Ca^2+^ buffers were undisturbed^44^. Therefore, we used BAM15 (10 μM) throughout this study to dissipate the proton gradient.

### Identifying off-target heat sources

We first measured the heat of mixing BAM15 in a saline bath without cells. As shown in Fig. 3b, the heat of mixing could elevate the temperature by a maximum of 1.2 ± 0.2 K (*n* = 6) over ~10 min. Remarkably, the observed temperature rise of ~1.2 K from heat of mixing without cells is comparable to a previously reported^19^ temperature change (~1 K) from mitochondrial thermogenesis (on COS7 cells) that ignored contribution from heat of mixing (Fig. 3a, Supplementary Fig. 5). To further characterize extraneous sources of heat, we measured the extracellular temperature rise due to BAM15 by placing the thermal probe immediately outside the membrane of a healthy neuron whose membrane potential was simultaneously recorded. At the extracellular level, the BAM15-triggered temperature elevations could be a result of bulk heating of all cells in the ganglion, and off-target effects from connective tissues, probes and saline. As shown in Fig. 3b, extracellular temperature increased to a maximum of 1.2 ± 0.6 K (*n* = 6), which then closely followed the response from heat of mixing. We thus observed that extraneous heat sources can result in extracellular temperature changes that decay slowly with a time constant, *τ*_e_ = 7.93 ± 3.62 min (shown in blue, Fig. 3b).

### Intracellular responses to proton uncoupling

**Fig. 4:**
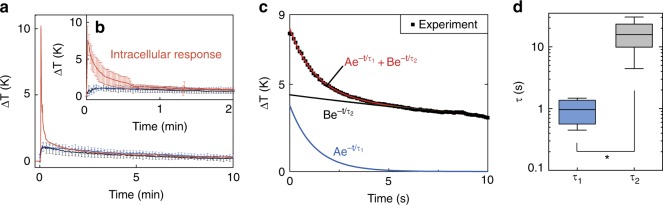
Identification of transient heat shock from mitochondrial proton uncoupling. **a** Representative data for an intracellular response with BAM15 is plotted (red) along with the control experiments (extracellular responses in blue, and heat of mixing in black). **b** A statistically averaged intracellular response from *n* = 6 trials is shown with the mean and the SD, and plotted along with control experiments. BAM15 responses begin at *t* = 0 min (*p* < 0.001 between intracellular and extracellular responses). **c** Representative Δ*T* measurement (*n* = 6) following BAM15 exposure is fit to a biexponential function (red). The intracellular temperature signals are a mix of two exponential decays: one with a short time constant, *τ*_1_, and other with a long time constant, *τ*_2_. **d** Time constants *τ*_1_ and *τ*_2_ (*p < 0.05) extracted from the measured Δ*T* data shown in Fig. 4b. The data are represented on a logarithmic scale

Once the thermal probe was inside the neuron (Fig. 3c, Supplementary Fig. 6), we measured the cell’s response to the protonophore BAM15 (10 μM). In Fig. 4a, a representative intracellular response (in red) to BAM15 shows a large temperature spike at time *t* = 0 min. In the inset, Fig. 4b, we show a statistically averaged intracellular response for the first 2 min following the addition of BAM15 (*n* = 6). We observed a transient pulse of 7.5 ± 2.0 K rise in intracellular temperatures after exposure to the proton uncoupler. On the other hand, control (Fig. 3b) and further experiments on vibration artifacts (Supplementary Fig. 7), and unhealthy cells (Supplementary Fig. 8), showed maximum Δ*T* ≲ 2.3 K over 10 min. From the latter, we conclude that the temperature changes of 7.5 ± 2.0 K in Fig. 4b (shown in red, *p* < 0.001) corresponded to specific intracellular responses to chemical proton uncouplers. We analyzed the relative magnitudes and time constants of the signals to understand the origin of the temperature response. Shown in Fig. 4c are representative data of the initial 10 s of thermal response to BAM15. We fit the data to a biexponential curve $Ae^{ - \frac{t}{{\tau _1}}} + Be^{ - \frac{t}{{\tau _2}}}$ to separate the short-term transients from the rest of the signal. The obtained time constants are shown in Fig. 4d. For the short-term component $Ae^{ - \frac{t}{{\tau _1}}}$, we found *A* = 4.8 ± 3.0 K with *τ*_1_ = 1.0 ± 0.4 s. The component with the longer time constant ($Be^{ - \frac{t}{{\tau _2}}}$) had *B* = 4.7 ± 0.9 K and *τ*_2_ = 16.6 ± 9.2 s. The time constants of intracellular responses (*τ*_1_, *τ*_2_) are an order of magnitude shorter than those associated with extracellular measurements (*τ*_e_).

## Discussion

We first discuss the possible source of the temperature component $Ae^{ - \frac{t}{{\tau _1}}}$. Any transients recorded in our experiments follow from transients in heat release and diffusion. Since the thermal time constant of the probe (~32 μs)^26^ is much smaller than *τ*_1_ and *τ*_2_, any heat release in the vicinity of the probe invokes an instantaneous response at the probe. We find the temporal response of $Ae^{ - \frac{t}{{\tau _1}}}$, with *τ*_1_ ≈ 1.0 s to be in close agreement with proton currents for which we calculated the time constant ($\tau _{H^ + }$) to be ~0.65 s from the data reported by Kirichok group^16^ (see Supplementary Fig. 1). Even though the duration of proton currents ($\tau _{H^ + }$) may vary across different cell lines, we find the time constant (*τ*_1_) to have a similar order of magnitude as $\tau _{H^ + }$ (Supplementary Fig. 1)^16^, suggesting that $Ae^{ - \frac{t}{{\tau _1}}}$ may arise from pmf dissipation in mitochondria, in close proximity to the probe.

While the temporal response (*τ*_1_) of the measured temperature change is consistent with predicted pmf dissipation time ($\tau _{H^ + }$), the magnitude of the temperature rise remains puzzling. A previous report^13^ predicted a maximum temperature rise rate of ~4.8 K/s during pmf dissipation but assumed adiabatic conditions and ignored heat diffusion from the mitochondria. We can estimate the heat release rate ($\dot Q_{\mathrm{p}}$) necessary to obtain the observed magnitude (Δ*T* = *A* ≈ 4.8 K) at our probe in the presence of heat diffusion. The temperature change occurred over a length scale of ~50 μm (Δ*x*) along the length of the probe that has a cross-sectional area (${\mathrm{\Delta }}y \cdot {\mathrm{\Delta }}z$~5 μm^2^). We can assume that temperature isotherms are spherically symmetric inside the cell given the similarity in the thermal conductivity (*k*) of the cell (~0.6 Wm^−1^ K^−1^)^45^ and the probe tip (*k*_*SiNx*_~0.8 Wm^−1^ K^−1^)^46^, respectively. This leads to an estimated (initial) heat flow through the probe of $\dot Q_{\mathrm{p}}\sim k_{SiNx}{\mathrm{\Delta }}y{\mathrm{\Delta }}z{\mathrm{\Delta }}T/{\mathrm{\Delta }}x$ ~400 nW. In comparison, the electrical heat at the onset of pmf dissipation from a single mitochondrion is ~20 pW ($\dot Q_{\mathrm{m}}\sim {\mathrm{\Delta }}p \cdot I_{H^ + }$)^13^, which may be dependent on the cell lines, and the proton pool. The density of mitochondria in axons^47^ can be as high as 0.6 μm^−2^. However, in the soma of *Aplysia* neurons, there can be a reticulum^48,49^ of several functionally fused mitochondria as well as numerous individual mitochondria^50,51^. A quantitative estimate of the typical number of mitochondria in the soma is not apparent^49^. Therefore, the gap between the predicted heat ($\dot Q_{\mathrm{m}}$) and the measured heat flow ($\dot Q_{\mathrm{p}}$) through the thermal probe could be due to the presence of numerous heat sources (mitochondria) that cannot be directly accounted for.

We now discuss the second component of the temperature rise. The slower decay component, $Be^{ - \frac{t}{{\tau _2}}}$ (with *τ*_2_ ≈ 16.6 s) can arise from a combination of glucose catabolism, heat of mixing, and a cumulative response of pmf dissipation in mitochondria at sites that either had delayed exposure to BAM15 or were more distant from the probe. The latter could be due to a heat diffusion time (*τ*_D_) of ~5 s (*τ*_D_~*L*^2^/*D*, *D* = 0.2 mm^2^ s^−1^)^45^ across a cell of ~1 mm diameter. Previous reports of large temperature changes over prolonged durations of ~5 min or more^19–24^ have been criticized on the theoretical implausibility of the prolonged temperature rise and the inaccuracies inherent in the non-invasive measurement techniques used^7–10,26^. Proton uncouplers like BAM15 and CCCP result in enhanced oxygen consumption and substrate oxidation that are typically sustained for ~20 min^41,52^ or more after exposure. Since we did not observe any appreciable intracellular temperature rise beyond ~33 s (2*τ*_2_), we either did not have prolonged substrate oxidation in the mitochondria of *Aplysia* neurons, or the temperature rise from substrate oxidation was negligible as suggested by Baffou et al.^7,10^.

The overall temperature rise observed in our experiment is on the same scale as those that have previously been shown to produce therapeutic heat shock responses by inducing heat shock proteins (Hsps)^53–56^. Hsps can protect against protein misfolding in disorders, such as Huntington’s disease, amyotrophic lateral sclerosis, and Parkinson’s disease^55^. Moreover, heat shock can protect neurons from programmed cell death by apoptosis^56^. Previously, heat shock responses have been observed due to ~4 K rise within 30 s of heating^57^ from a microscopy stage. In our work, the observed transient temperature rise ~7.5 K occurs intracellularly, which gives an added spatiotemporal advantage over external heating. Since the magnitude (~7.5 K) and time constant (2*τ*_2_~33 s) are comparable to the required conditions^57^ for a heat shock response, the impact of stimulated pmf dissipation on secondary heat shock responses remains an open question for further study.

In this work, we used the proton uncoupler BAM15 to induce the mitochondrial proton gradient dissipation, which otherwise may not occur under normal physiological circumstances in *Aplysia* neurons. Possible targets for future investigations are natural thermogenic cells such as inguinal, epididymal, and brown fat cells^17^ in which the uncoupling protein UCP1 is endogenously expressed. Such adipocytes may occasionally dissipate proton gradients using UCP1s, depending on the demand for heat production^5^. However, the thermal probe used in this work is too large (~5 μm wide) for measurements in adipocytes (≲50 μm). Moreover, to measure the mitochondrial proton motive force (Δ*p*), fluorescent probes for both the mitochondrial membrane potential (Δ*ψ*_m_) and the mitochondrial pH difference are required^58^. Existing fluorescent probes have undesirable effects that can result in electron transport chain toxicity^58^, and temperature^14^ and plasma membrane potential (Δ*ψ*_p_) dependence^58^ of the measured mitochondrial potential Δ*ψ*_m_. Thus, future studies that can combine nonperturbative proton motive force (Δ*p*) measurement with an electrically inert, smaller (<1 μm) invasive intracellular thermal probe may provide more information on endogenous heat release through pmf dissipation by UCP1.

In summary, temperature measurements using an inert and high-speed microthermal probe reveal fundamental thermogenesis mechanisms that were previously missed in time-averaged fluorescence-based techniques^19–24^. We observe a transient thermal shock of ~7.5 K at the onset of stimulated proton uncoupling. Upon decomposing the signal into individual components, we detect a component of the signal with a large magnitude (~4.8 K), and a small time constant (~1.0 s) that correspond well to proton diffusion time scale during proton transport across mitochondrial inner membrane. As the observed transient thermal shock (~4.8 K) dominates the steady-state processes, transient pmf dissipation stands out as a viable candidate for therapies targeting stimulated non-shivering thermogenesis. Further studies with biological UCPs and different concentrations of chemical proton uncouplers could reveal additional insights for physiologically relevant pmf dissipation rates during endogenous homeostatic thermogenesis.

## Methods

### Culture dish preparation

We utilized neurons from the abdominal ganglion of *Aplysia californica*. Animals were obtained from the NIH/University of Miami National Resource for Aplysia Facility (Miami, FL) and housed at the University of Illinois in a 200-gallon closed marine system maintained at 12–13 °C. Animals were anesthetized through injections of 330 mM MgCl_2_ and the abdominal ganglion was dissected out. The abdominal ganglion was placed in a culture dish filled with room-temperature saline (composition, in mM: 420 NaCl, 10 KCl, 25 MgCl_2_, 25 MgSO_4_, 10 CaCl_2_, 10 HEPES buffer, pH = 7.5) and secured using insect pins. After microdissection and desheathing, the cells were accessible to the electrodes. The ventral aspect of the ganglion was carefully dissected to provide access to the neurons of interest. We prepared 1 mM solutions of BAM15 (Sigma-Aldrich) in 100 μL DMSO, which would form a final concentration of ~10 μM when added to the culture dish containing saline ~10 mL. We ensured consistency in temperatures of BAM15 and saline using external thermocouple probes (Omega Type-K).

### Microelectrode preparation

Intracellular voltage recordings were made using borosilicate microelectrodes filled with 3 M KCl and pulled to a resistance of 11–16 MΩ. Intracellular microelectrodes were connected to an intracellular amplifier (Model 1600, A-M Systems, Sequim, WA), which were in turn connected to a data acquisition system (PowerLab 8/30, ADInstruments, Dunedin, New Zealand). Real-time voltage recordings were digitized and recorded in LabChart 7.3 (ADInstruments) at sampling rates of 20 kHz.

### Intracellular temperature measurements

To measure intracellular temperature changes, we first penetrated the target neuron with a voltage electrode. We then penetrated the same neuron with the thermal probe. In this process, the neuron released an injury discharge as shown in Fig. 3c and Supplementary Fig. 6, and slowly returned to a healthy resting potential as shown in Supplementary Fig. 6b, and during *t* < 1 min in Fig. 3a. In conjunction with visual inspection of the probe tip’s position, this served as an additional confirmation that the thermal probe successfully entered the target cell. A Keithley nanovoltmeter (2182A) was used to measure the Seebeck voltage, which is calibrated to be read as temperature changes. The sampling frequency was limited to 10 Hz by the external electronics.

### Statistics and reproducibility

Information on statistics and number of trials are shown in the corresponding figure or figure captions. Where necessary, data points are representative of mean values with s.d. shown as error bars. *p*-values were estimated using *t*-test.

### Reporting summary

Further information on research design is available in the Nature Research Reporting Summary linked to this article.

## Supplementary information

Supplementary Information

Description of supplementary items file

Supplementary Data 1

Reporting Summary

## Data Availability

Data that support the findings of this study are available from the corresponding authors upon reasonable request. Raw data used to generate the plots can be found in Supplementary Data 1 file accompanying this paper.
