# Supplementary material for: Transient heat release during induced mitochondrial proton uncoupling
Source: Commun Biol. 2019 Jul 26;2:279. doi: 10.1038/s42003-019-0535-y (PMC6659641; doi:10.1038/s42003-019-0535-y)
Supplement: Supplementary file 1 — Supplementary Information [file 42003_2019_535_MOESM1_ESM.docx]

**SUPPLEMENTARY INFORMATION**


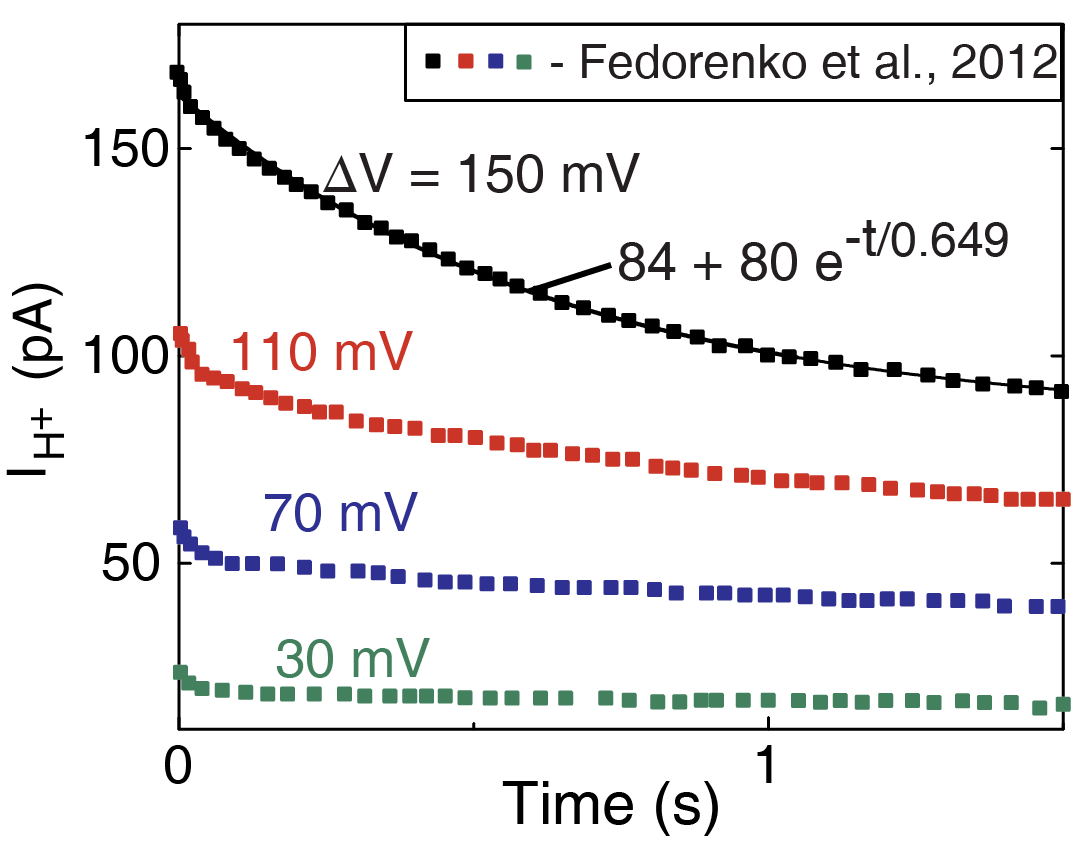


**Supplementary Figure 1**: Measured proton currents from mitochondrial voltage patch clamp are plotted from experiments performed by Fedorenko *et al*^1^. At *t* = 0 s, the set voltage V is changed from 0 to 30 mV, 70 mV, 110 mV, and 150 mV. The voltage step generates a proton current that slowly saturates over time. We fit the current for the most relevant data set ($\Delta$*V* = 150 mV, because mitochondrial proton motive force^2, 3^ is ~ 200 mV) to an exponential decay function with offset, since current need not be zero at *t* → ∞. We find that the proton current has a time constant ~0.65 s.


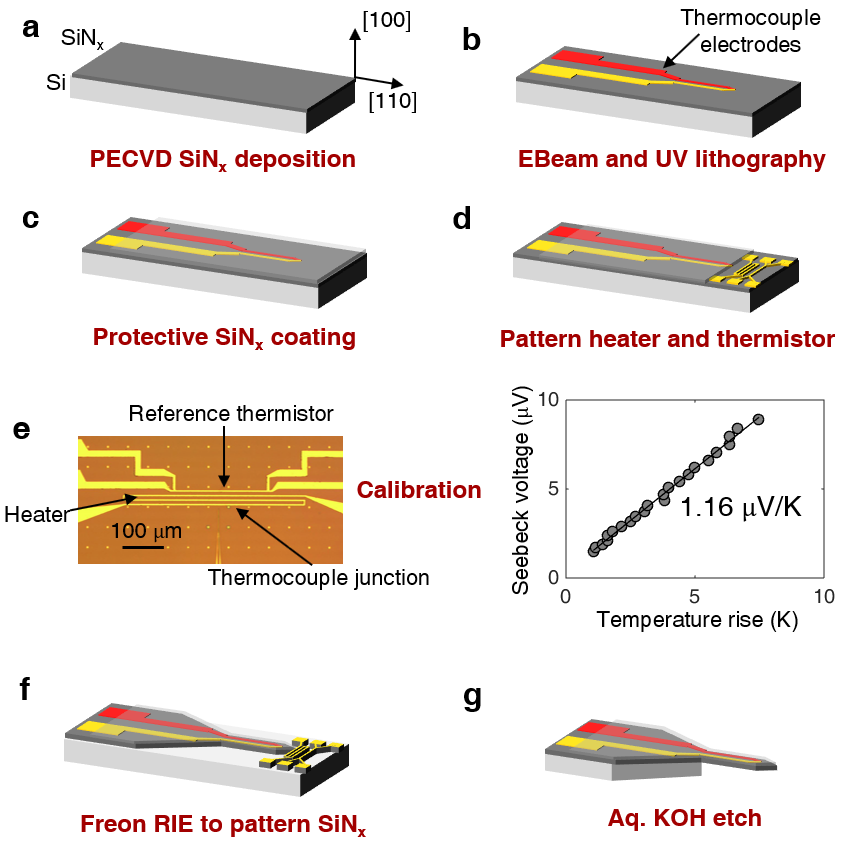


**Supplementary Figure 2**: a) We begin fabrication of the thermal probe with a PECVD silicon nitride coating. b) A combination of e-beam and UV lithography techniques are used for patterning the thermocouple metal lines. c) The thermocouple is coated with a protective nitride layer. d) UV and e-beam lithography techniques are used for patterning the heater, and reference thermistor metal lines. e) Calibration is done in a vacuum cryostat. The Seebeck voltage of the thermocouple is calibrated against known temperature rises. We calculated the calibration accuracy to be $\pm$54 mK for 300 K $\pm$ 10 K using finite element methods^4^. f) Freon RIE transfers a UV lithography pattern to the underlying SiN_x_ to define the probe’s profile. g) An aq. KOH etch is done to suspend the calibrated probe. The fabrication procedure discussed here is an updated version of that presented in our previous work^4^.


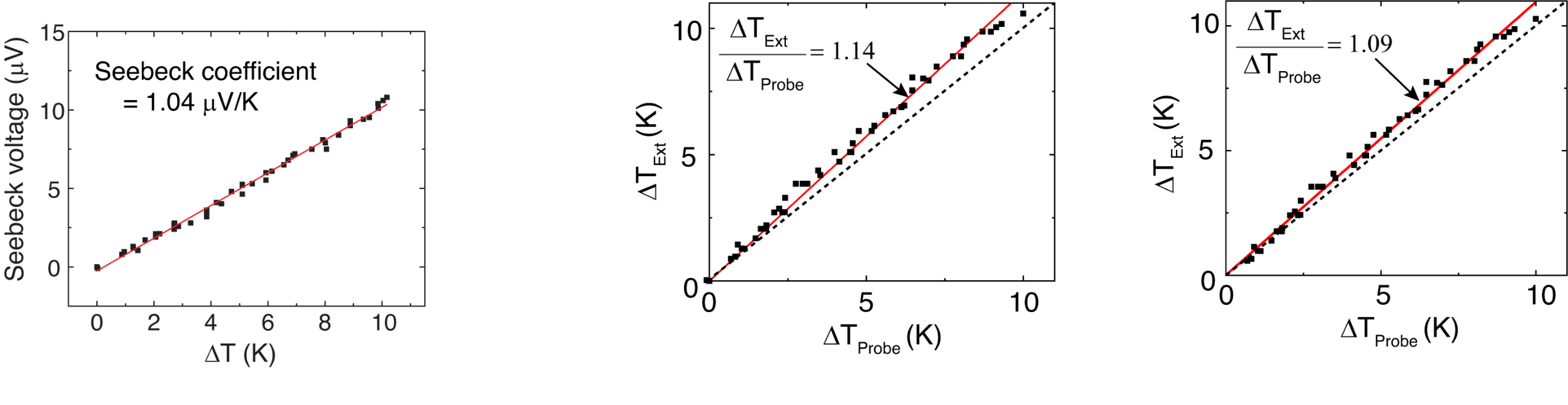


**Supplementary Figure 3**: Using a heated microscopy stage, we measured the temperature changes using the microthermal probe ($\Delta T_{Probe}$) placed inside a neuron of buccal ganglion and also an external thermistor ($\Delta T_{Ext}$) placed in the saline bath ~1 cm away from the ganglion. An omega thermistor (TH-44032-40-T) was used in conjunction with a recording device (Measurement Computing USB-TEMP) to measure $\Delta T_{Ext}$. We heated the culture dish by 10 K over a period of 1 hour in steps of 2-3 K. The Seebeck voltage from the microthermal probe yielded $\Delta T_{Probe}$, using prior calibration (Supplementary Figure 2e). The bath’s temperature rise ($\Delta T_{Ext}$) was obtained from the external thermistor. We repeated the measurements three times with different step sizes; all data points are shown on the graph. The red line is a linear fit to the measured data, whereas the black dashed line corresponds to a slope of 1. The temperature measurement from the external thermistor is different from the microthermal probe’s reading by ~9.5%. The difference, in magnitude, is as large as ~300 mK at the largest temperature rise of ~10 K. We attribute this difference to arise from convection currents inside the bath.


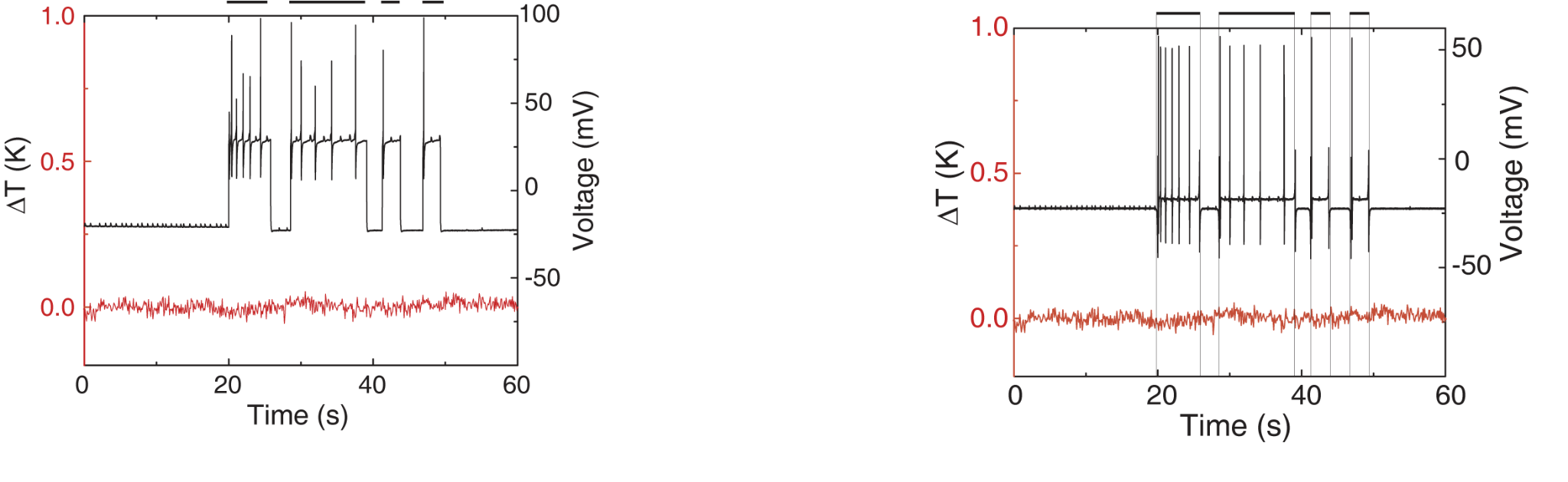


**Supplementary Figure 4**: We tested the common mode noise response of the thermal probe by changing a neuron’s potential with respect to ground. We placed both the thermal probe and the sharp microelectrode inside a neuron. The voltage plot shows the microelectrode reading that was high-pass infinite impulse response filtered to remove offsets from the electrode resistance. Starting at *t* = 20 s, we repeatedly depolarized the neuron by passing a constant current though the microelectrode; durations of the current are represented using dashed lines above the plot. This can result in a common mode signal on the two electrodes of the thermal probe that is also inside the cell. If the thermal probe was not insulated enough from the common mode signal in the neuron, a corresponding signal in the form of a voltage differential would be observed from the Nanovoltmeter^5^. For instance, a common mode signal of ~100 mV can result in 0.1 $\mu V$ apparent Seebeck voltage^5^ that corresponds to ~0.1 K. Since, we observe < 20 mK temperature changes during depolarization and action potentials in the neuron, we can assume that the ~300 nm silicon nitride electrically insulates the probe from the typical electrical activity in neurons during our experiments.


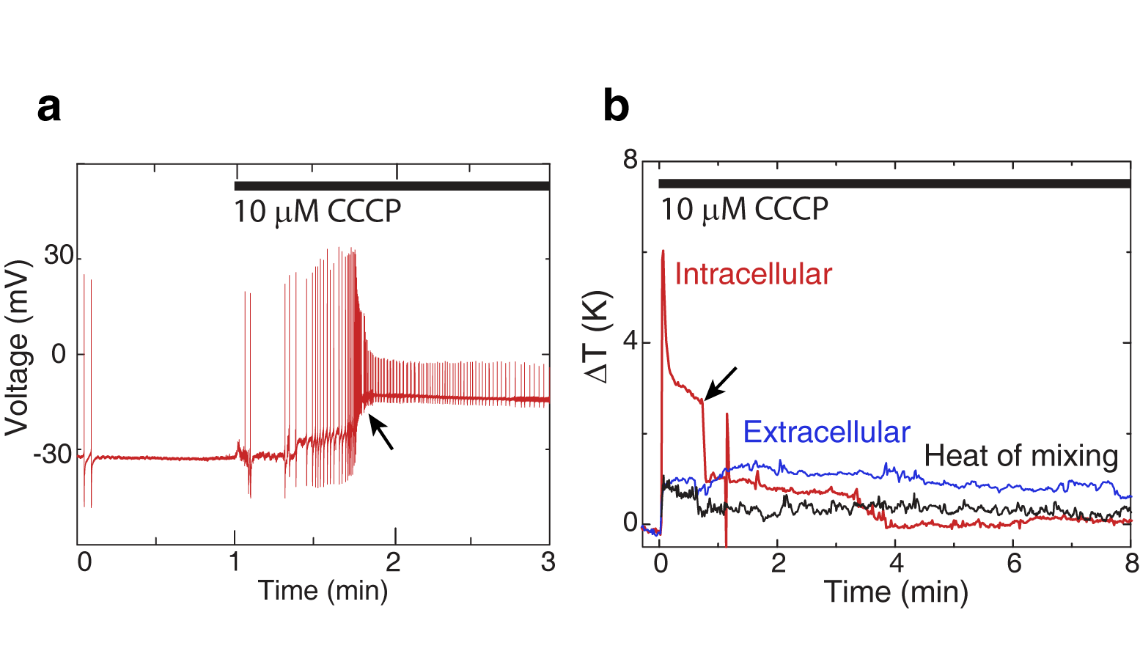


**Supplementary Figure 5**: a) Representative intracellular voltage recording from a KCl electrode during the addition of CCCP (representation of n=2 experiments). The neuron was penetrated by the thermal probe at-least 30 min before CCCP addition. b) Temperature changes measured following CCCP exposure at *t* = 0 min. The initial intracellular temperature response ~6.1 K rise is higher than that of extracellular and saline response (heat of mixing). We mark an apparent depolarization event in black arrows that roughly occurred 1 min after CCCP exposure in both figures. This apparent depolarization could be from a combination of endogenous depolarization of the cell, and from electrode and thermal probe movement due to contraction of smooth muscle in the connective tissue. To avoid such off-target activities^6, 7, 8^ caused by the widely used proton uncoupler CCCP, we opted instead to use BAM15^6^, a less cytotoxic proton uncoupler, to dissipate the mitochondrial proton motive force. Further, the maximum temperature rise of ~ 6.1 K from CCCP appears to be comparable to our experiments from BAM15 (Fig. 4a), suggesting that heat from Ca^2+^ currents is negligible in comparison to proton currents.


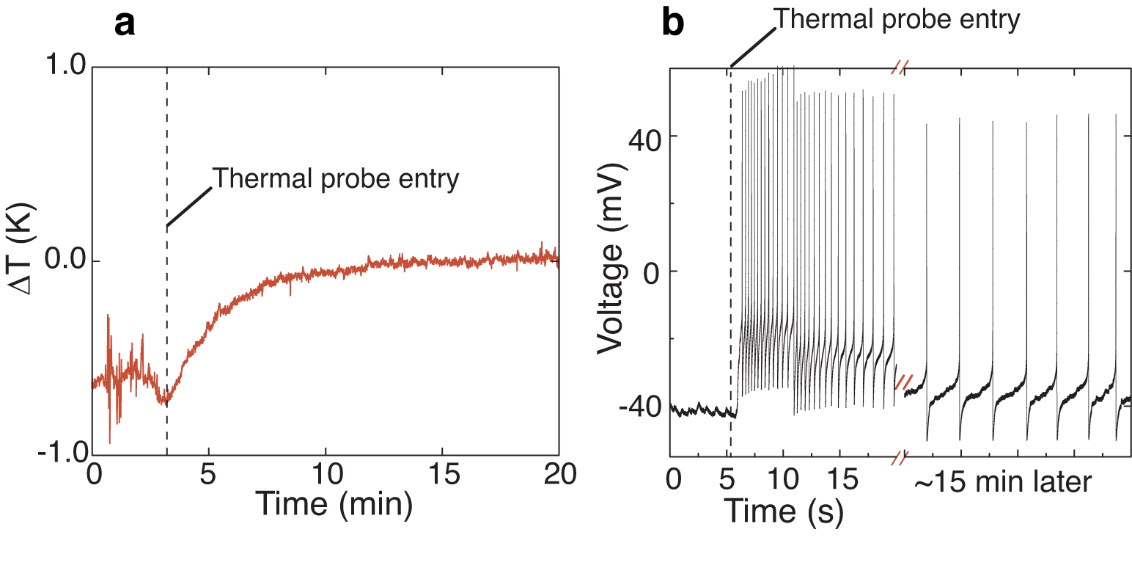


**Supplementary Figure 6**: a) We show the temperature response from the microthermal probe as it penetrates the cell. After penetration into the cell, it takes about ~10 min for the temperature to stabilize. The rather long stabilization time (~10 min) can be attributed to the membrane injury response. b) The neuron produces high-frequency discharge following the thermal probe entry. After ~15 min, the discharge frequency reduces, and the resting membrane potential is partly recovered. All the experiments reported in this work were performed only after the temperature and electrical activity stabilized within the cell.


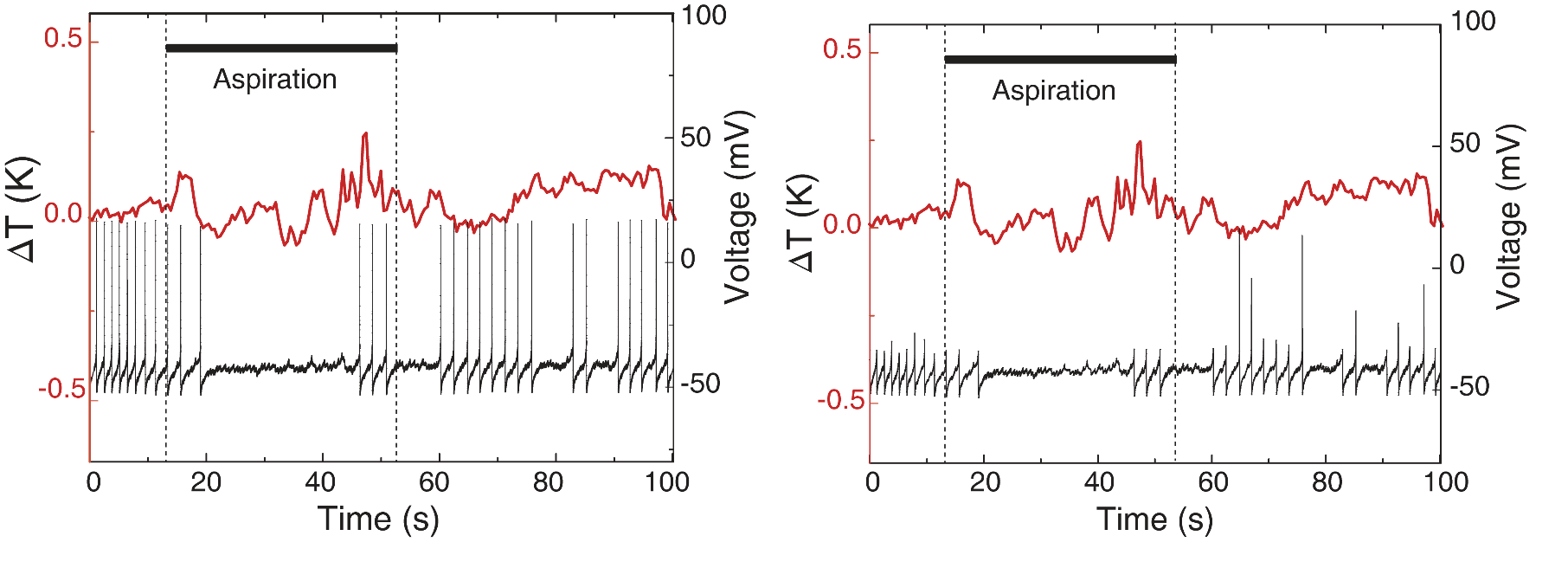


**Supplementary Figure 7**: We emulate the injection and stirring of BAM15 by repeated aspirations of saline with an empty Pasteur pipette to observe the possible thermal artifacts arising from stirring. Temperature changes were measured inside a neuron using the micro-fabricated thermal probe. We also measure the intracellular electrical activity of the cell using a sharp microelectrode. Shown here is a representative response from stirring. No significant temperature or electrical activity changes were observed from repeated experiments ($\Delta T$<0.5 K, n=6).


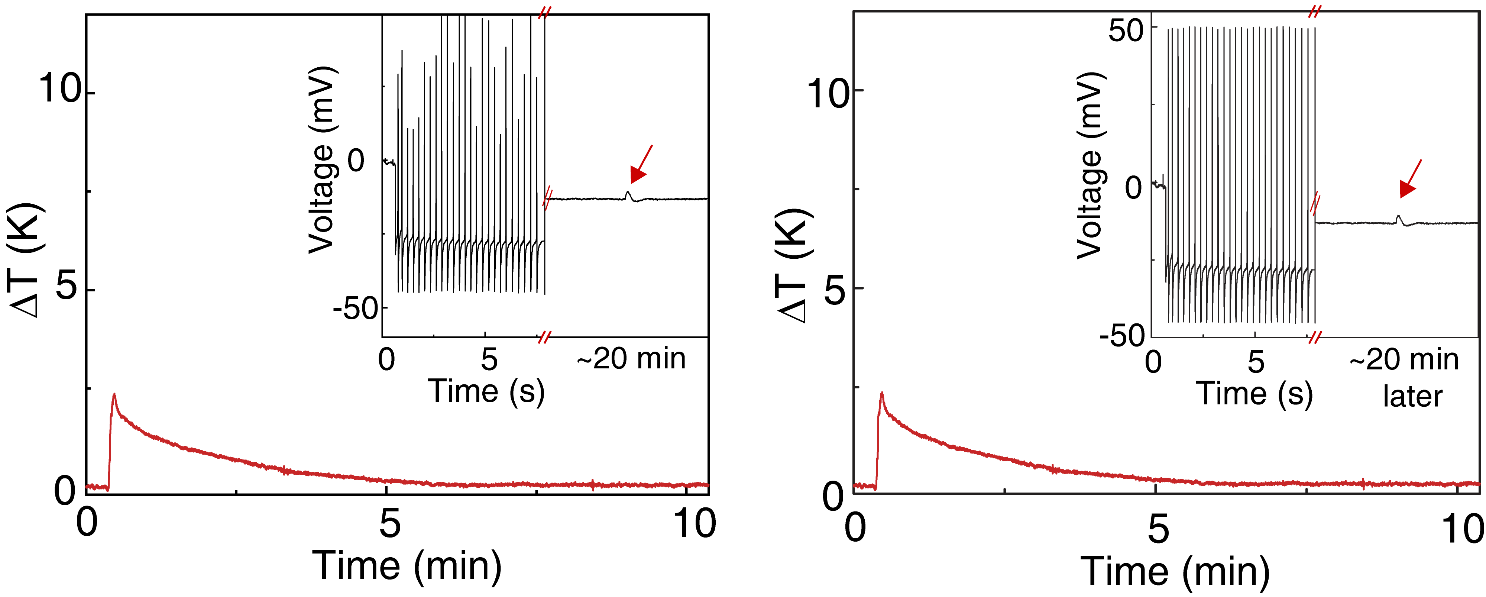


**Supplementary Figure 8**: Intracellular response to BAM15 from a less viable cell shows a thermal shock of ~2.3 K, which is significantly lower than the mean $\Delta$*T* ~7.5 K shown in Fig. 4. This ganglion was kept refrigerated for ~3 days after removal from the host *Aplysia californica*. In the inset, the initial half of the electrophysiological recording shows that the resting potential is ~ -25 mV when the microelectrode entered the neuron. The latter half of the recording corresponds to conditions following thermal probe entry and before BAM15 addition. The neuron occasionally showed excitatory postsynaptic potential (shown in red arrow) but no action potentials, and the resting potential increased to ~ -13 mV. The electrophysiological recordings show that this neuron is less viable than usual. All the experiments reported in Fig. 4 were from neurons that had a resting potential <-30 mV, with action potential magnitudes ~70 mV (representative data in Figs. 3a and Supplementary Figure 6b), which is typical for neurons that were tested within four hours after removal from the host *Aplysia*.

**Supplementary References**

1. Fedorenko, A., Polina V. Lishko, and Y. Kirichok, *Mechanism of Fatty-Acid-Dependent UCP1 Uncoupling in Brown Fat Mitochondria.* Cell, 2012. **151**(2): p. 400-413.
2. Kang, J.-S., *Theoretical model and characteristics of mitochondrial thermogenesis.* Biophysics Reports, 2018. **4**(2): p. 63-67
3. Korzeniewski, B. and W. Froncisz, *An extended dynamic model of oxidative phosphorylation.* Biochimica et Biophysica Acta (BBA) - Bioenergetics, 1991. **1060**(2): p. 210-223.
4. Rajagopal, M.C., et al., *Fabrication and Characterization of Thermocouple Probe for Use in Intracellular Thermometry.* Sensors and Actuators A: Physical, 2018
5. Keithley, J.F., *Low level measurements: for effective low current, low voltage, and high impedance measurements*. 1984: Keithley Instruments
6. Kenwood, B.M., et al., *Identification of a novel mitochondrial uncoupler that does not depolarize the plasma membrane.* Molecular Metabolism, 2013. **3**(2): p. 114-123.
7. Summers, D.W., A. DiAntonio, and J. Milbrandt, *Mitochondrial Dysfunction Induces Sarm1-Dependent Cell Death in Sensory Neurons.* The Journal of Neuroscience, 2014. **34**(28): p. 9338.
8. Yang, J.-H., et al., *Apoptotic cell death of cultured salamander photoreceptors induced by cccp: CsA-insensitive mitochondrial permeability transition.* Journal of cell science, 2001. **114**(9): p. 1655-1664.
