# Supplementary material for: Transient heat release during induced mitochondrial proton uncoupling
Source: Commun Biol. 2019 Jul 26;2:279. doi: 10.1038/s42003-019-0535-y (PMC6659641; doi:10.1038/s42003-019-0535-y)
Supplement: Supplementary file 2 — Description of supplementary items file [file 42003_2019_535_MOESM2_ESM.docx]

**Description of Additional Supplementary Files**

**File Name**: Supplementary Data 1.xlsx

**Description**:  Contains the raw data for all the main figures in an Excel format.
