# Supplementary material for: Transient heat release during induced mitochondrial proton uncoupling
Source: Commun Biol. 2019 Jul 26;2:279. doi: 10.1038/s42003-019-0535-y (PMC6659641; doi:10.1038/s42003-019-0535-y)
Supplement: Supplementary file 4 — Reporting Summary [file 42003_2019_535_MOESM4_ESM.pdf]

nature research

Corresponding author(s): Sanjiv Sinha, and Rhanor Gillette

Last updated by author(s): Jun 23, 2019

Reporting Summary

Nature Research wishes to improve the reproducibility of the work that we publish. This form provides structure for consistency and transparency in reporting. For further information on Nature Research policies, see Authors & Referees and the Editorial Policy Checklist.

Statistics

For all statistical analyses, confirm that the following items are present in the figure legend, table legend, main text, or Methods section.

n/a	Confirmed	
[ ]	[x]	The exact sample size (*n*) for each experimental group/condition, given as a discrete number and unit of measurement
[ ]	[x]	A statement on whether measurements were taken from distinct samples or whether the same sample was measured repeatedly
[ ]	[x]	The statistical test(s) used AND whether they are one- or two-sided<br>*Only common tests should be described solely by name; describe more complex techniques in the Methods section.*
[ ]	[x]	A description of all covariates tested
[x]	[ ]	A description of any assumptions or corrections, such as tests of normality and adjustment for multiple comparisons
[ ]	[x]	A full description of the statistical parameters including central tendency (e.g. means) or other basic estimates (e.g. regression coefficient) AND variation (e.g. standard deviation) or associated estimates of uncertainty (e.g. confidence intervals)
[ ]	[x]	For null hypothesis testing, the test statistic (e.g. *F*, *t*, *r*) with confidence intervals, effect sizes, degrees of freedom and *P* value noted<br>*Give P values as exact values whenever suitable.*
[x]	[ ]	For Bayesian analysis, information on the choice of priors and Markov chain Monte Carlo settings
[x]	[ ]	For hierarchical and complex designs, identification of the appropriate level for tests and full reporting of outcomes
[x]	[ ]	Estimates of effect sizes (e.g. Cohen's *d*, Pearson's *r*), indicating how they were calculated

Our web collection on statistics for biologists contains articles on many of the points above.

Software and code

Policy information about availability of computer code

Data collection	National Instruments LabVIEW 2017 for temperature data, and LabChart 7.3 (ADInstruments) for voltage recordings.
Data analysis	MATLAB, Python, Origin Pro, and Microsoft Excel were used for data analysis, processing, and statistical estimations.

For manuscripts utilizing custom algorithms or software that are central to the research but not yet described in published literature, software must be made available to editors/reviewers. We strongly encourage code deposition in a community repository (e.g. GitHub). See the Nature Research guidelines for submitting code & software for further information.

Data

Policy information about availability of data

All manuscripts must include a data availability statement. This statement should provide the following information, where applicable:

- Accession codes, unique identifiers, or web links for publicly available datasets
- A list of figures that have associated raw data
- A description of any restrictions on data availability

We provide the raw data for the main figures in Supplemental Data file. Further data supporting this study can be provided upon reasonable requests

Field-specific reporting

Please select the one below that is the best fit for your research. If you are not sure, read the appropriate sections before making your selection.

[x] Life sciences [ ] Behavioural & social sciences [ ] Ecological, evolutionary & environmental sciences

For a reference copy of the document with all sections, see nature.com/documents/nr-reporting-summary-flat.pdf

Life sciences study design

All studies must disclose on these points even when the disclosure is negative.

Sample size	No statistical method was used to predetermine the sample size. Sample sizes are reported in all measurements in the corresponding figures and figure captions.
Data exclusions	Intracellular temperature data were only utilized from cells that produced an injury discharge in the voltage readings while inserting the thermal probe. If injury discharge was not elicited during thermal probe entry, we discard the data as the thermal probe may not have entered the cell.
Replication	All experiments were repeated. All attempts at replication that produced an injury discharge were successful and were included in the reported data
Randomization	For each experiment, we used a different nerve cell (chosen randomly) from the abdominal ganglion of Aplysia. For control experiments with no cell, we randomized the location of the intracellular probe within the saline bath.
Blinding	The analysis of the experiments are defined by the described methods, and do not require blinding for objective analysis.

Reporting for specific materials, systems and methods

We require information from authors about some types of materials, experimental systems and methods used in many studies. Here, indicate whether each material, system or method listed is relevant to your study. If you are not sure if a list item applies to your research, read the appropriate section before selecting a response.

Materials & experimental systems

n/a	Involved in the study
☒	☐ Antibodies
☒	☐ Eukaryotic cell lines
☒	☐ Palaeontology
☐	☒ Animals and other organisms
☒	☐ Human research participants
☒	☐ Clinical data

Methods

n/a	Involved in the study
☒	☐ ChIP-seq
☒	☐ Flow cytometry
☒	☐ MRI-based neuroimaging

Animals and other organisms

Policy information about studies involving animals; ARRIVE guidelines recommended for reporting animal research

Laboratory animals	We utilized neurons from the abdominal ganglion in Aplysia californica. Specimens were obtained from the NIH/University of Miami National Resource for Aplysia Facility (Miami, FL) and maintained at the University of Illinois in a 200-gallon closed marine system maintained at 12-13° C
Wild animals	We did not involve wild animals
Field-collected samples	We did not involve samples collected from field
Ethics oversight	The standardized protocol for handling Aplysia is mentioned in the Methods section. No further ethical guidance was required

Note that full information on the approval of the study protocol must also be provided in the manuscript.
